# Level of functioning and symptoms in children and adolescents with early-onset psychosis - baseline characteristics of participants in the OPUS YOUNG trial

**DOI:** 10.1192/j.eurpsy.2025.323

**Published:** 2025-08-26

**Authors:** N. K. Andersen, N. Larsen, C. F. Nielsen, T. L. Andersen, M. S. Madsen, A. K. Pagsberg, T. O. Y. group

**Affiliations:** 1 Child and Adolescent Mental Health Center, Copenhagen University Hospital –Mental Health Services CPH; 2 Department of Clinical Medicine, University of Copenhagen, Copenhagen, Denmark

## Abstract

**Introduction:**

Level of functioning is often severely affected in individuals diagnosed with psychosis. Often, negative symptoms have been proposed as an important treatment target to improve functioning. Most research on the topic is conducted in adult samples, but the functional impairments associated in adolescents with schizophrenia spectrum disorders might differentiate from adults. A detailed understanding of the functional impairments and its association to clinical symptoms is important to develop meaningful and effective treatments adjusted to the needs of this young patient group.

**Objectives:**

The aim of this study is 1) to explore functional capacity across different domains of function, and 2) to examine associations between functioning and clinical symptoms in youth with early-onset psychosis.

**Methods:**

The study sample consists of 290 children and adolescent aged 12-17 years with early-onset psychosis included in the OPUS YOUNG trial. All participants’ level of functioning was assessed with the Personal and Social Performance Scale (PSP), ranged 0-100. The level of psychosis symptoms was assessed with Scale for the Assessment of Positive Symptoms in Schizophrenia (SAPS) and Scale for the Assessment of Negative Symptoms in Schizophrenia (SANS). Data on affective symptoms, duration of untreated psychosis, cognitive functioning, quality of life, and substance use was also collected.

**Results:**

The participants had a mean age of 15.6 (SD=1.6) years and 72% were females. Mean PSP total score was 49.9 (SD=13.8) and most participants had manifest to marked difficulties on the domains Socially useful activities, Personal and social relationships, and Self-care. Few participants had impairments in the domain of Disturbing and aggressive behavior. On average, levels of positive symptoms, negative symptoms, disorganized symptoms, and affective symptoms were of mild severity. Preliminary correlation analyses showed that all symptom categories were statistically significantly associated with PSP total score, and negative symptoms showed the strongest association.

**Conclusions:**

Level of functioning was markedly impaired across most domains while symptom levels were of mild severity in most participants. We suggest that improvement of functioning should be a primary treatment target in parallel with symptom alleviation of this patient group.

**Disclosure of Interest:**

None Declared

